# Biodiversity and Activity of Gut Fungal Communities across the Life History of *Trypophloeus klimeschi* (Coleoptera: Curculionidae: Scolytinae)

**DOI:** 10.3390/ijms19072010

**Published:** 2018-07-10

**Authors:** Guanqun Gao, Jing Gao, Chunfeng Hao, Lulu Dai, Hui Chen

**Affiliations:** 1 College of Forestry, Northwest A&F University, Yangling 712100, China; ggqun@nwafu.edu.cn (G.G.); sxllgaojing@nwafu.edu.cn (J.G.); dailulu@nwafu.edu.cn (L.D.); 2 Tianjin Forestry Pest Control and Quarantine Station, Tianjin 300000, China; wjx2017@nwafu.edu.cn; 3 State Key Laboratory for Conservation and Utilization of Subtropical Agro-Bioresources, College of Forestry and Landscape Architecture, South China Agricultural University, Guangzhou 510642, China

**Keywords:** *Trypophloeus klimeschi*, life stages, intestinal fungal, fungal communities, integrated pest management

## Abstract

We comprehensively investigated the biodiversity of fungal communities in different developmental stages of *Trypophloeus klimeschi* and the difference between sexes and two generations by high throughput sequencing. The predominant species found in the intestinal fungal communities mainly belong to the phyla Ascomycota and Basidiomycota. Fungal community structure varies with life stage. The genera Nakazawaea, Trichothecium, Aspergillus, Didymella, Villophora, and Auricularia are most prevalent in the larvae samples. Adults harbored high proportions of Graphium. The fungal community structures found in different sexes are similar. Fusarium is the most abundant genus and conserved in all development stages. Gut fungal communities showed notable variation in relative abundance during the overwintering stage. Fusarium and Nectriaceae were significantly increased in overwintering mature larvae. The data indicates that Fusarium might play important roles in the survival of *T. klimeschi* especially in the overwintering stage. The authors speculated that *Graphium* plays an important role in the invasion and colonization of *T. klimeschi*. The study will contribute to the understanding of the biological role of the intestinal fungi in *T. klimeschi*, which might provide an opportunity and theoretical basis to promote integrated pest management (IPM) of *T. klimeschi*.

## 1. Introduction

According to current statistics, more than 10% of insects in nature interact with symbiotic microorganisms [1]. The interaction between symbiotic fungi and bark beetles has also been studied extensively [2,3,4,5,6,7]. The vast majority of bark beetles are closely related to symbiotic fungi at various stages of development, and some bark beetles directly use the fungal fruiting bodies or fungal hyphae colonized in the gallery as a food source [2,8]. A variety of symbiotic fungi, from the microecological point of view of the bark beetles, make the larvae more advantageous than other xylophagous insects. The bark beetles use intestinal microbiotas to improve the utilization of plant carbon and nitrogen nutrition, which is more difficult for insects to decompose, thereby improving the constraints of the bark beetles on overcoming the nutrient-poor factors of food sources and ensuring their development and reproduction [9,10,11]. *Trypophloeus klimeschi* Eggers (Coleoptera, Curculionidae, Scolytinae) was first recorded in the Kyrgyz Republic, which borders Xinjiang Province in China [12]. Following an outbreak in 2003 in Xinjiang Province, *T*. *klimeschi* spread rapidly to the adjacent areas. The widespread outbreak of this beetle has caused huge economic, ecological, and social losses in China’s northwest shelter forest. The insect is now found in Dunhuang, where it has been identified as *T*. *klimeschi* by morphology [12,13]. This is the first systematic survey of fungal communities across the life cycle of *T*. *klimeschi.* The previous research on the intestinal fungal diversity of insects was conducted mainly through traditional methods such as culture separation and morphological identification [14]. This provides an important basis for the composition and species diversity of insect gut fungal, but it is inevitably incomplete in the description of insect gut microbes. According to statistics, approximately 99% of the microorganisms in nature are not culturable [15], but molecular biology technology can make up for this limitation. Molecular biology methods allow for the subsequent sequencing and analysis of the DNA to characterize fungal species composition and abundance [16].

There have been many reports on the research of intestinal micro-organisms with bark beetles, such as the intestinal microflora of some bark beetles showed differences in different geographic environment [17,18]. However, there are few studies on the differences in the entire development stage of the bark beetles, including difference in sexes. Recently, the symbiotic relationship between insects and their intestinal microbiota has attracted widespread attention from scholars around the world. Studies have shown that symbiotic microbes play a very important role in the invasion, settlement, spawning, development, reproduction, and other roles in developmental and life cycle stages of bark beetles [19,20,21,22]. Concurrently, a better understanding of the symbiosis formed by an insect and its colonizing microorganisms could be useful to improve insect control, use and development [17,23]. Through clarifying the composition of insect gut fungi, scientists can further study the role of gut fungi in the host physiology.

High throughput sequencing technology is used to study the fungal community structure and diversity dynamics at different developmental stages, different generations, and between *T*. *klimeschi* adult males and females. The results reveal the interaction between symbiotic microorganisms and *T. klimeschi* and provide a theoretical basis for the development of new biological control technologies.

## 2. Results

### 2.1. Overview of Sequencing Analysis

**Figure 1:**
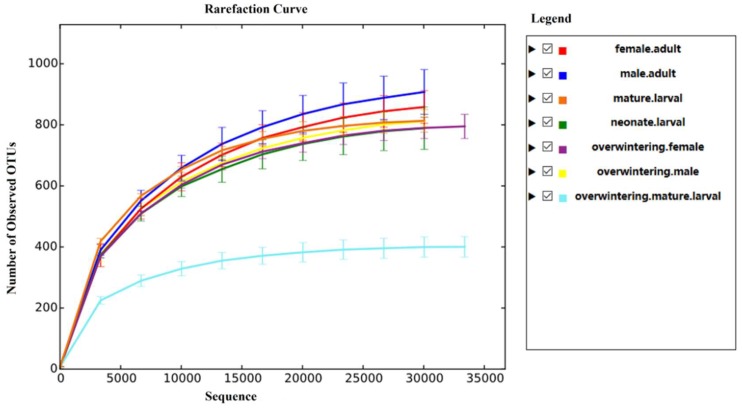
The rarefaction curve in each sample (Note: OTU: Operational Taxonomic Unit).

The proportion which equaled the number of high quality sequences/valid sequences was over 97% in each development stage (Table 1). Briefly, raw sequencing reads with exact matches to the barcodes were assigned to respective samples and identified as valid sequences. The low-quality sequences were filtered through the following criteria: sequences that had a length of <150 bp, sequences that had average Phred scores of <20, sequences that contained ambiguous bases, and sequences that contained mononucleotide repeats of >8 bp. Following sequence trimming, quality filtering, and removal of chimeras, the number of sequences per sample (overwintering mature larval, overwintering female adult, overwintering male adult, neonate larvae, mature larvae, female adult, and male adult) was 41,015 ± 2088, 47,239 ± 2773, 43,208 ± 1023, 45,476 ± 1562, 46,214 ± 1804, 45,491 ± 2324, 43,728 ± 1163, respectively. The rarefaction curves indicated that species representation in each sample approached the plateau phase, and it was unlikely that more fungi would be detected with additional sequencing efforts (Figure 1).

### 2.2. The Diversity and Community Structure of Fungal Diversity in Different Development Stages of T. klimeschi

#### 2.2.1. Fungal Diversity in Different Development Stages

**Figure 2:**
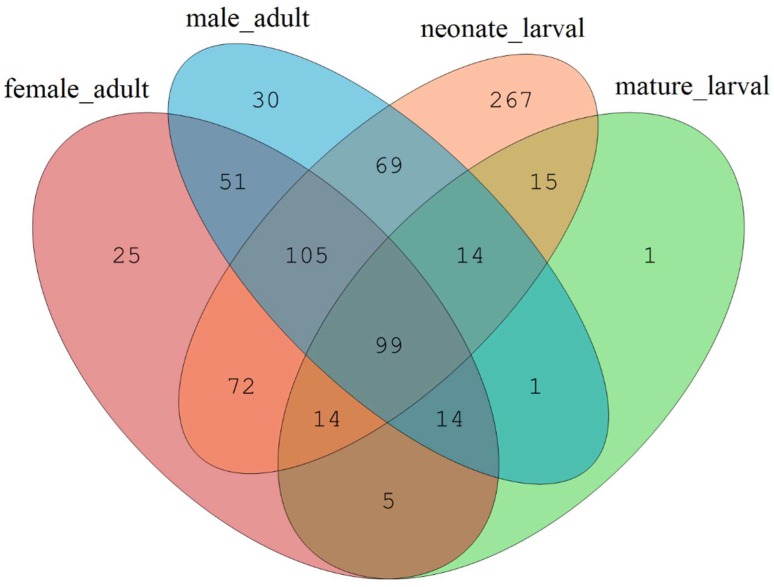
Venn diagram representing the distribution of the fungal OTUs in different development stages of *T. klimeschi*.

*T. klimeschi* undergoes complete metamorphosis: larvae and adults differ greatly in form and function. This study divided the relatively long larval stage into neonate larval and mature larval stages. The high-quality sequences were clustered into different OTUs (Operational Taxonomic Units) by the UPARSE pipeline at a 3% dissimilarity level. The authors found abundant fungi persisted in all development stages of *T. klimeschi* but, the diversity and structure of the *T. klimeschi*—associated fungal community varied significantly across host development stages. A Venn diagram was used to compare the similarities and differences between the communities in different development stages (Figure 2). Subsequent to sequence standardization, the fungal community in the neonate larvae was more diverse than the community identified in the mature larvae. Fungal community richness further increased in adults. Chao and ACE (Abundance-based Coverage Estimator) index values suggested that the fungal community richness decreased from neonate larvae to mature larvae then increased from mature larvae to adult (Table 2).

#### 2.2.2. Fungal Community Composition and Structure Succession Analysis

**Figure 3:**
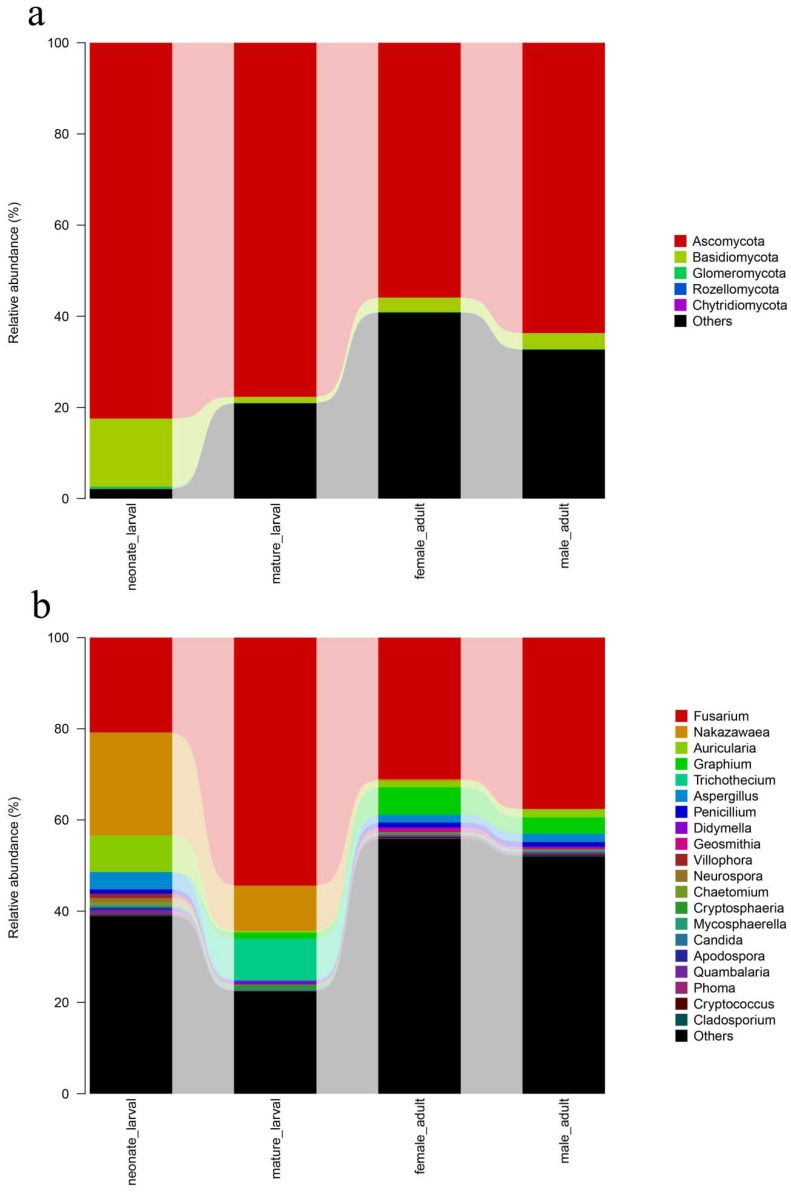
Fungal community structure variation in different development stages of *T. klimeschi* at the phylum level and the genus level. (**a**) phylum level; (**b**) genus level.

To identify gut fungal community structure succession in different development stages, the ITS (Internal Transcribed Spacer) sequences were classified at the phylum, class, order, and family levels. The taxonomic analysis of sequences revealed that the most prevalent phylum in all development stages was Ascomycota (69.933 ± 13.828% of the sequence) and Basidiomycota (6.152 ± 7.928% of the sequence). There were notable trends and changes in the relative abundance of the different fungi taxa in different development stages (Figure 3a). The relative abundances of Ascomycota were significantly decreased after eclosion (ANOVO: *F* = 4.850, df = 3, *p* = 0.033). Basidiomycota were richer in neonate larval than during other life stage (ANOVO: *F* = 4.751, df = 3, *p* = 0.035).

#### 2.2.3. Clustering Patterns of Samples in Different Development Stages

**Figure 4:**
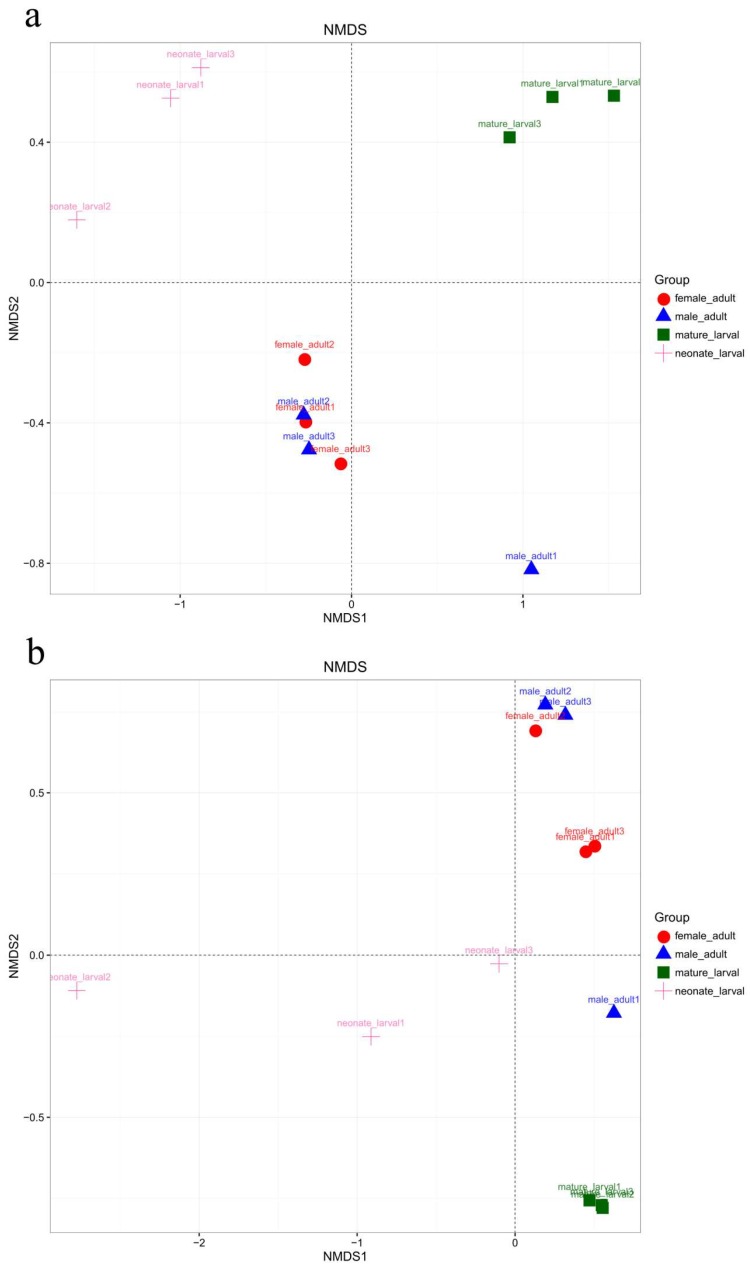
Nonmetric multidimensional scaling analysis of the Bray–Curtis dissimilarity index of the fungal community OTUs (≥97% identity) in different development stages of *T. klimeschi* based on Illumina sequencing of ITS genes. (**a**) Unweighted; (**b**) Weighted.

The authors compared community structures between samples using Nonmetric multidimensional scaling (NMDS) that revealed the development pattern in each life stage for the unweighted and weighted UniFrac distances (Figure 4). According to the unweighted UniFrac NMDS, neonate larvae and mature larvae formed a unique cluster, separated from the adults. According to principal coordinates (NMDS1 and NMDS2), the differences in fungal communities were great between neonate larvae, mature larvae and adults, while the differences were small in adult females compared with adult males. According to the weighted UniFrac NMDS, the neonate larval, mature larval and adults were separated and clustered based on NMDS1and NMDS2.

#### 2.2.4. Differences Between Samples in Different Development Stages

Differences in the community composition among different development stages were tested using the Duncan’s test for multiple comparisons. The genera Nakazawaea, Trichothecium, Aspergillus, Didymella, and Villophora which belonged to the Ascomycota; and Auricularia which belonged to Basidiomycota were more prevalent in the larvae samples (*p* ≤ 0.05). Nakazawaea, Aspergillus, Villophora, and Auricularia were abundant and active in neonate larvae, while Trichothecium and Didymella significantly increased in mature larvae (*p* ≤ 0.05). Adults harbored high proportions of Graphium which belonged to Ascomycota (*p* ≤ 0.05). The author also found Fusarium, which belonged to Ascomycota, persisted through metamorphosis and did not have a significant change in relative content (*p* > 0.05). Furthermore, Fusarium was the most abundant genus in different development stages (Figure 3b) (See Table A1 in Appendix A).

### 2.3. The Diversity and Community Structure of Fungal Diversity in Different Generations

#### 2.3.1. Fungal Diversity in Different Generations

**Figure 5:**
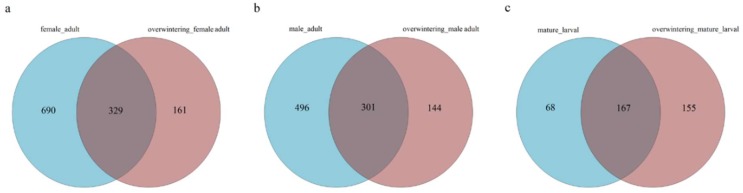
Venn diagram representing the distribution of the fungal OTUs in different generations of *T. klimeschi.* (**a**) Adult females vs. overwintering adult females; (**b**) adult males vs. overwintering adult males; (**c**) mature larvae vs. overwintering mature larvae.

Due to the significant environmental differences between the two generations, the authors quantified the composition and structure of gut fungal communities of the first generation and overwintering generation. The high-quality sequences were clustered into different OTUs by the UPARSE pipeline at a 3% dissimilarity level. A Venn diagram was used to compare the similarities and differences between the communities in different generations (Figure 5). Chao and ACE index values suggested that there were significant differences in the fungal community richness between the two generations in larvae. This trend was also true for Simpson and Shannon index values (Table 3). The fungal community in larvae of the overwintering generation was more diverse than the community identified in larval of the first generation. However, there were no significant differences of the fungal community richness in adults between two generations.

#### 2.3.2. Fungal Community Composition and Structure Succession Analysis

**Figure 6:**
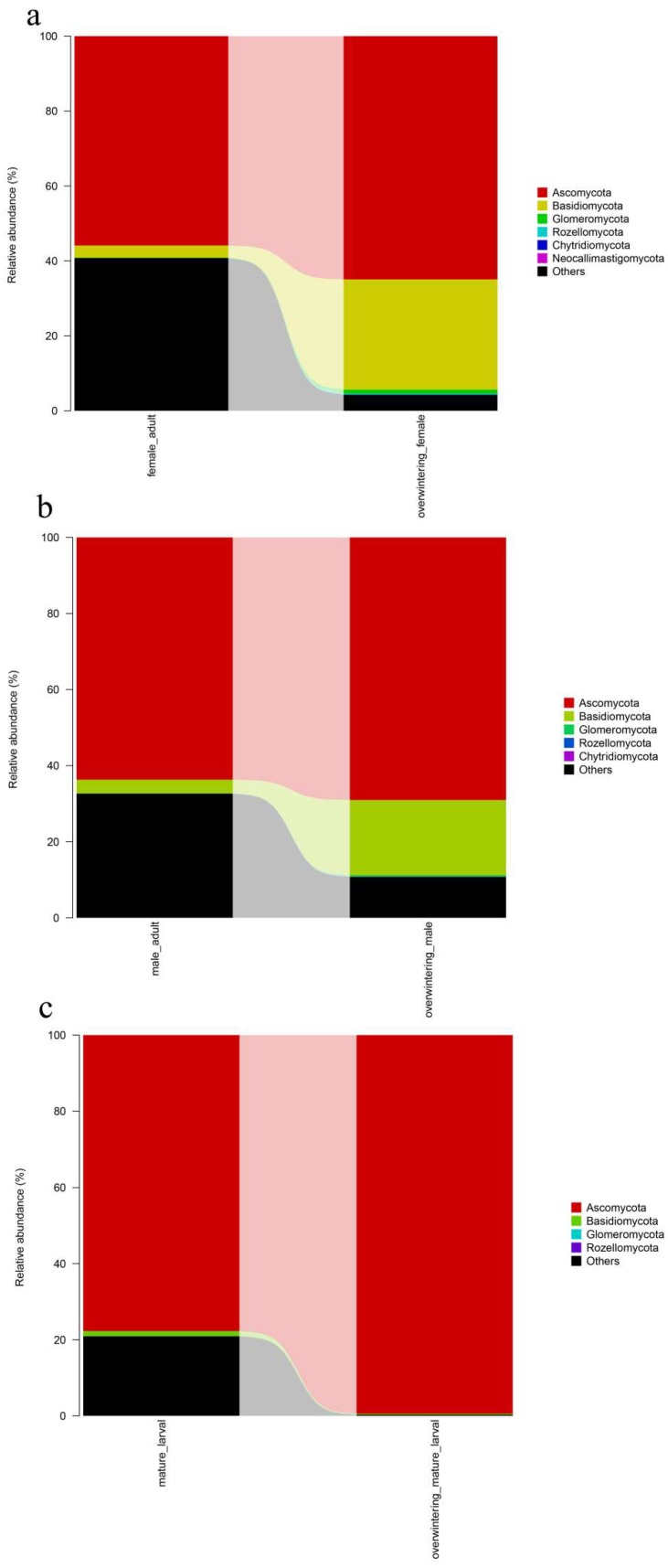
Fungal community structure variation in different generations of *T. klimeschi* at the phylum level. (**a**) Adult females vs. overwintering adult females; (**b**) adult males vs. overwintering adult males; (**c**) mature larvae vs. overwintering mature larvae.

To identify gut fungal community structure succession in different generations, the ITS sequences were classified at the phylum, class, order, and family levels. There were notable trends and changes in the relative abundance of the different fungal taxa in different generations (Figure 6). The relative abundance of Ascomycota was significantly increased in the overwintering generation (*p* ≤ 0.05). (See Table A2 in Appendix A).

#### 2.3.3. Clustering Patterns of Samples in Different Generation

**Figure 7:**
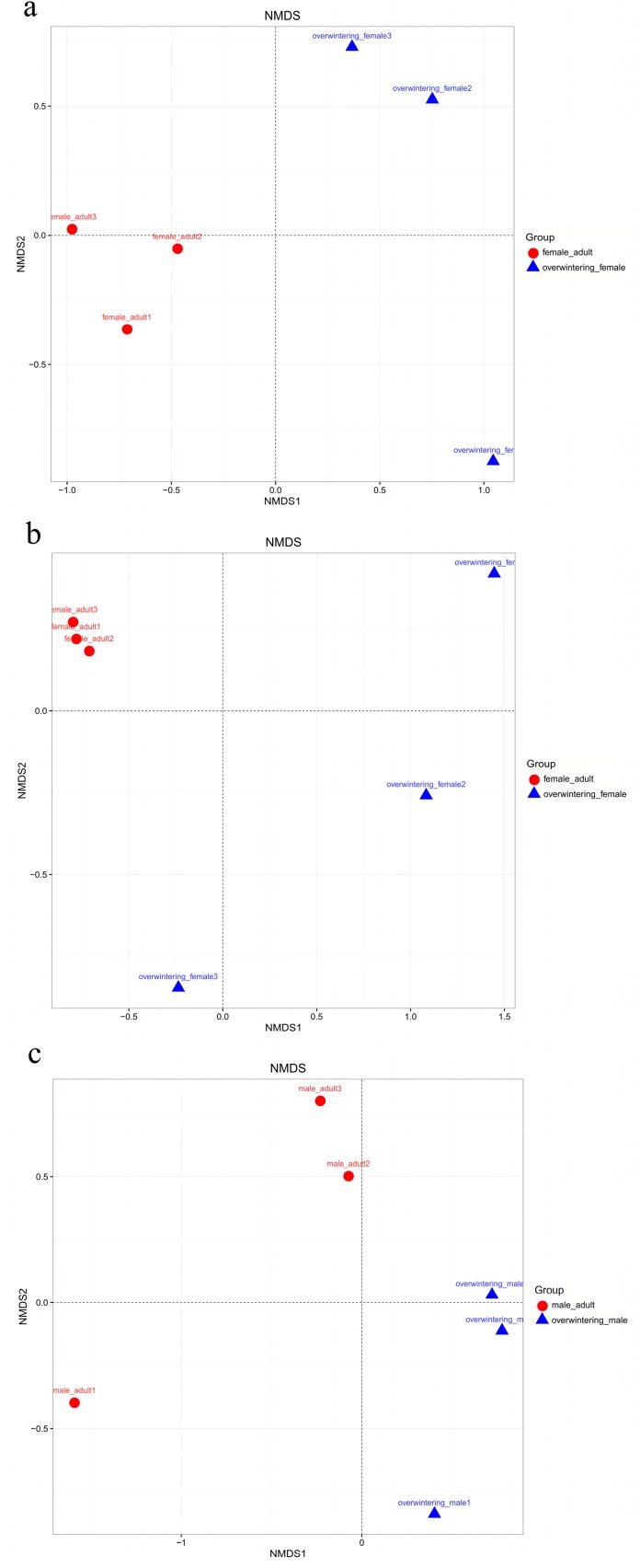

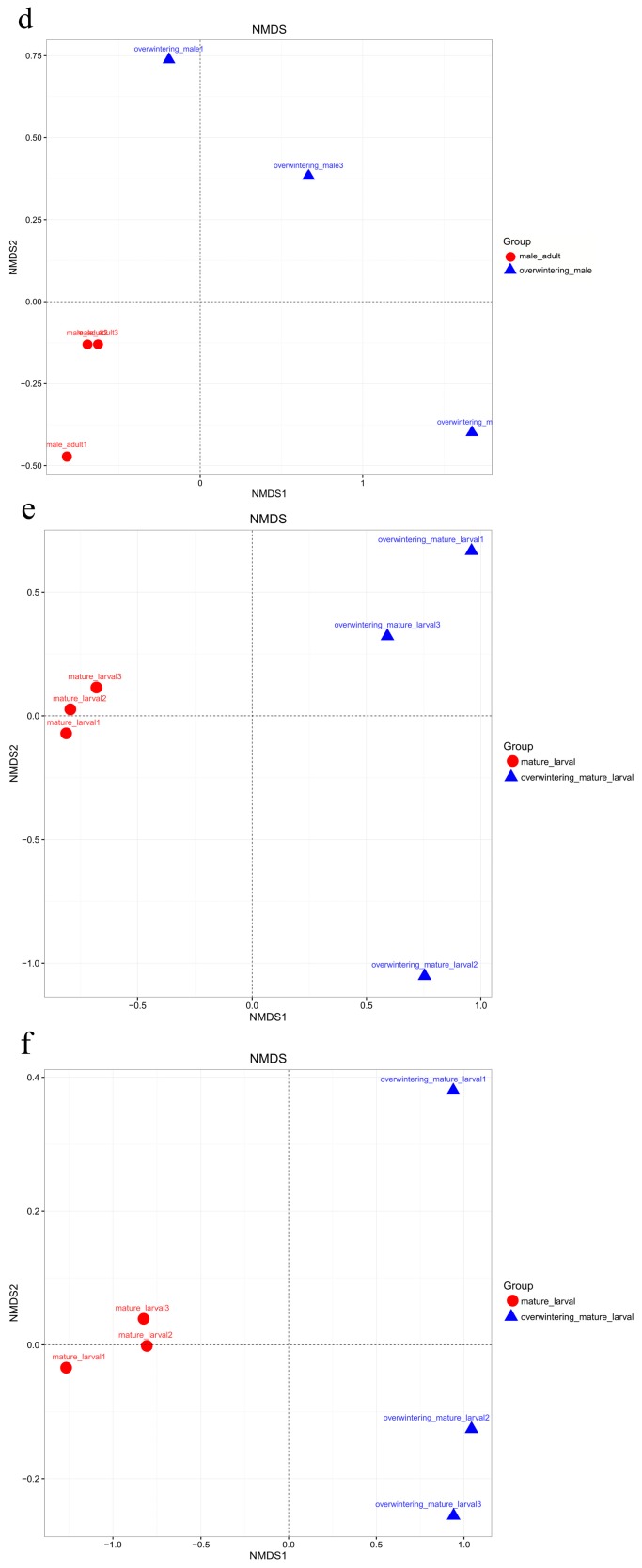
Nonmetric multidimensional scaling analysis of the Bray–Curtis dissimilarity index of the fungal community OTUs (≥97% identity) in different generations of *T. klimeschi* based on Illumina sequencing of ITS genes. Adult females vs. overwintering adult females: (**a**) Unweighted; (**b**) Weighted; adult males vs. overwintering adult males: (**c**) Unweighted; (**d**) Weighted; mature larvae vs. overwintering mature larvae: (**e**) Unweighted; (**f**) Weighted.

The authors compared community structures between samples using NMDS that revealed the development pattern in different generations for the unweighted and weighted UniFrac distances (Figure 7A,B). According to the unweighted UniFrac NMDS, the overwintering generation formed a unique cluster, separated from the first generation. According to principal coordinates (NMDS1 and NMDS2), the differences in fungal communities were great between the overwintering generation and first generation. This trend was also true for the weighted UniFrac NMDS.

#### 2.3.4. Differences Between Samples in Different Generations

**Figure 8:**
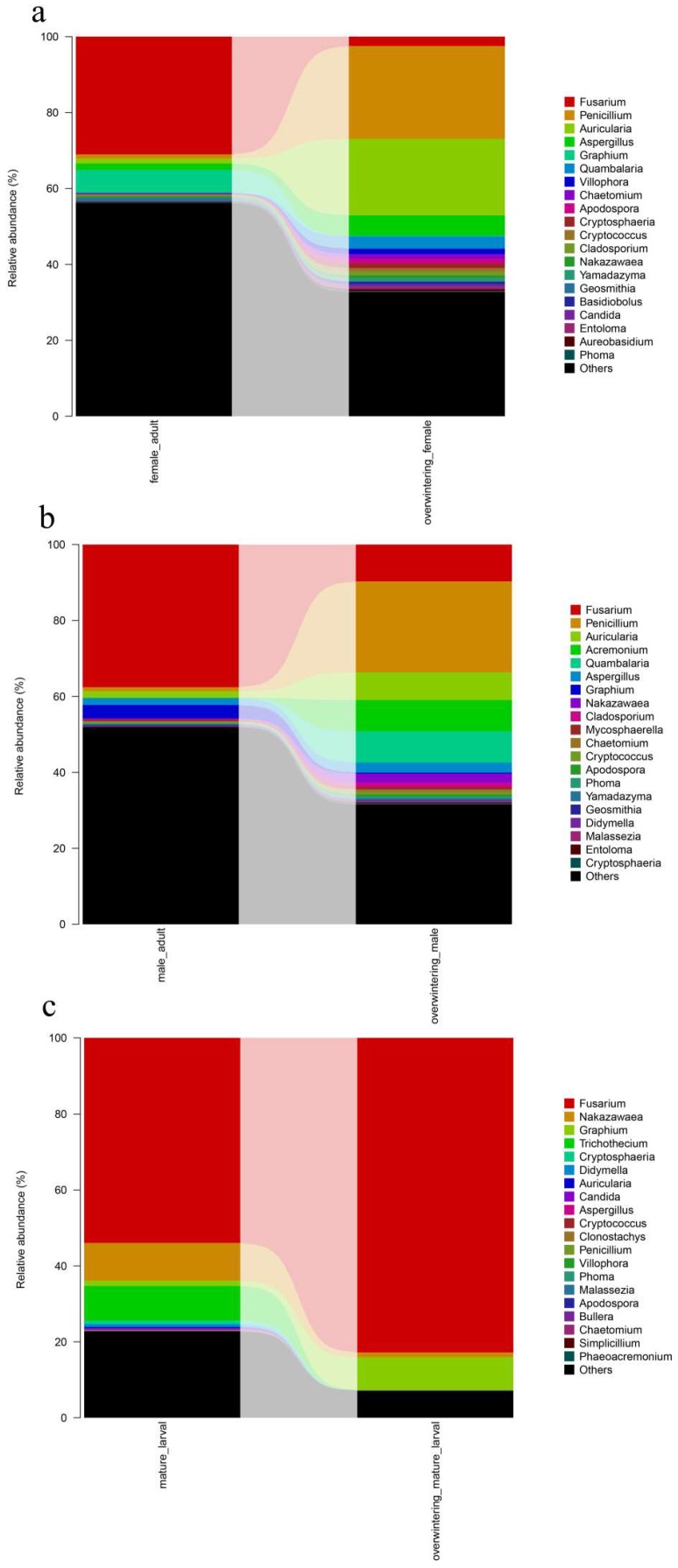
Fungal community structure variation in different generations of *T. klimeschi* at the genus level. (**a**) Adult females vs. overwintering adult females; (**b**) adult males vs. overwintering adult males; (**c**) mature larvae vs. overwintering mature larvae.

Differences in the community composition among different generations were tested using the Duncan’s test for multiple comparisons. The diversity and composition of the *T. klimeschi*-associated fungal community varied substantially between the two generations. Regarding adults, Aspergillus, which belonged to the Ascomycota, was significantly increased in the overwintering generation (*p* ≤ 0.05). The pest overwintered in the form of mature larvae. The authors found that the fungal community during the overwintering stage varied substantially. Fusarium, which belongs to the Ascomycota, was significantly increased in overwintering mature larvae (*p* ≤ 0.05), whereas Nakazawaea, which belongs to the Ascomycota, decreased (*p* ≤ 0.05). Trichothecium and Cryptosphaeria were absent in overwintering mature larvae. Unidentified_Nectriaceae was only appeared in overwintering mature larvae. (Figure 8) (See Table A3 in Appendix A).

### 2.4. The Diversity and Community Structure of Fungal Diversity between Males and Females

#### 2.4.1. Fungal Diversity in Different Sexes

**Figure 9:**
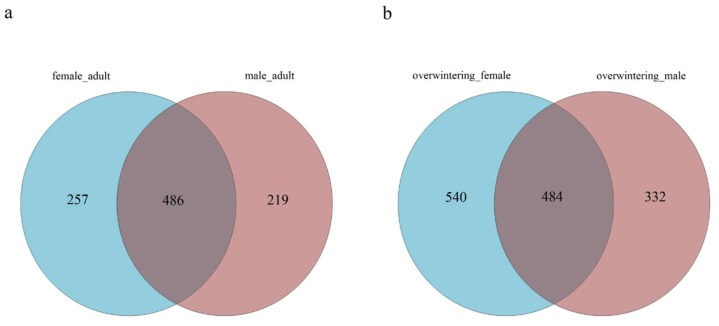
Venn diagram representing the distribution of the fungal OTUs in different sexes of. (**a**) Adult females vs. adult males; (**b**) overwintering adult females vs. overwintering adult males.

Considering the difference between adult males and adult females, the internal fungal communities associated with mature adults of each sex were studied separately. A Venn diagram was used to compare the similarities and differences between the communities in different sexes (Figure 9). Chao and ACE index values suggested that there were no significant differences of the fungal community richness in different sexes. This trend was also true for the Simpson and Shannon index values (Table 4).

#### 2.4.2. Fungal Community Composition and Structure Succession Analysis.

**Figure 10:**
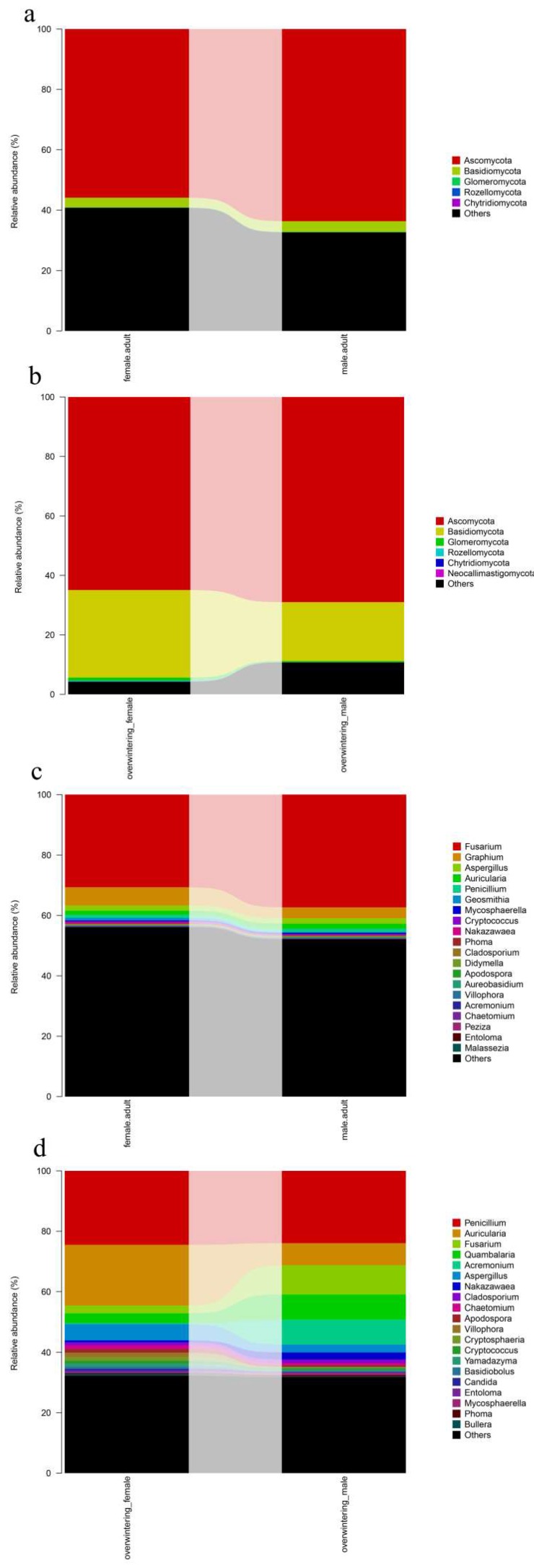
Fungal community structure variation in different sexes of *T. klimeschi* at the phylum level and genus level. (**a**,**b**) Phylum level; (**c**,**d**) genus level2.4.3. Clustering Patterns of Samples in Different Sexes.

To identify gut fungal community structure succession in different sexes, the ITS sequences were classified at the phylum, class, order, and family levels. There were notable trends and changes in the relative abundance of the different fungi taxa in different sexes (Figure 10a,b). There were no significant differences of gut fungal community between females and males (see Table A4 in Appendix A).

**Figure 11:**
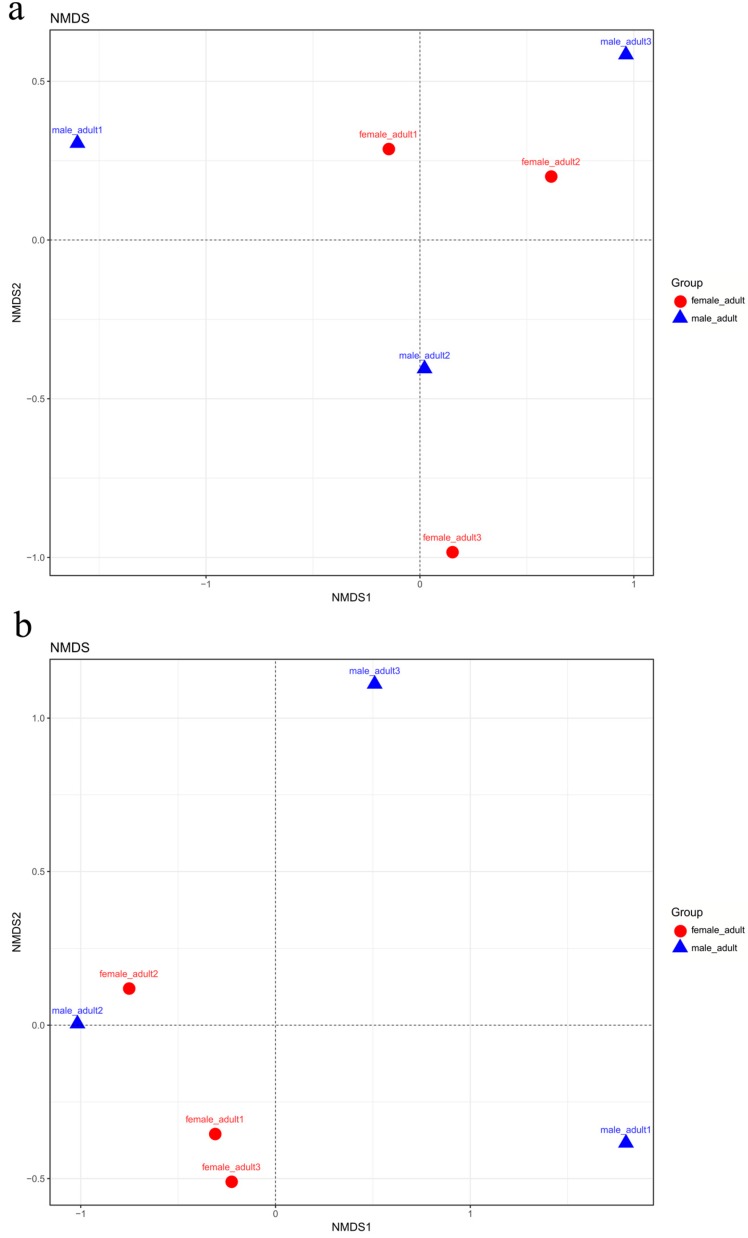

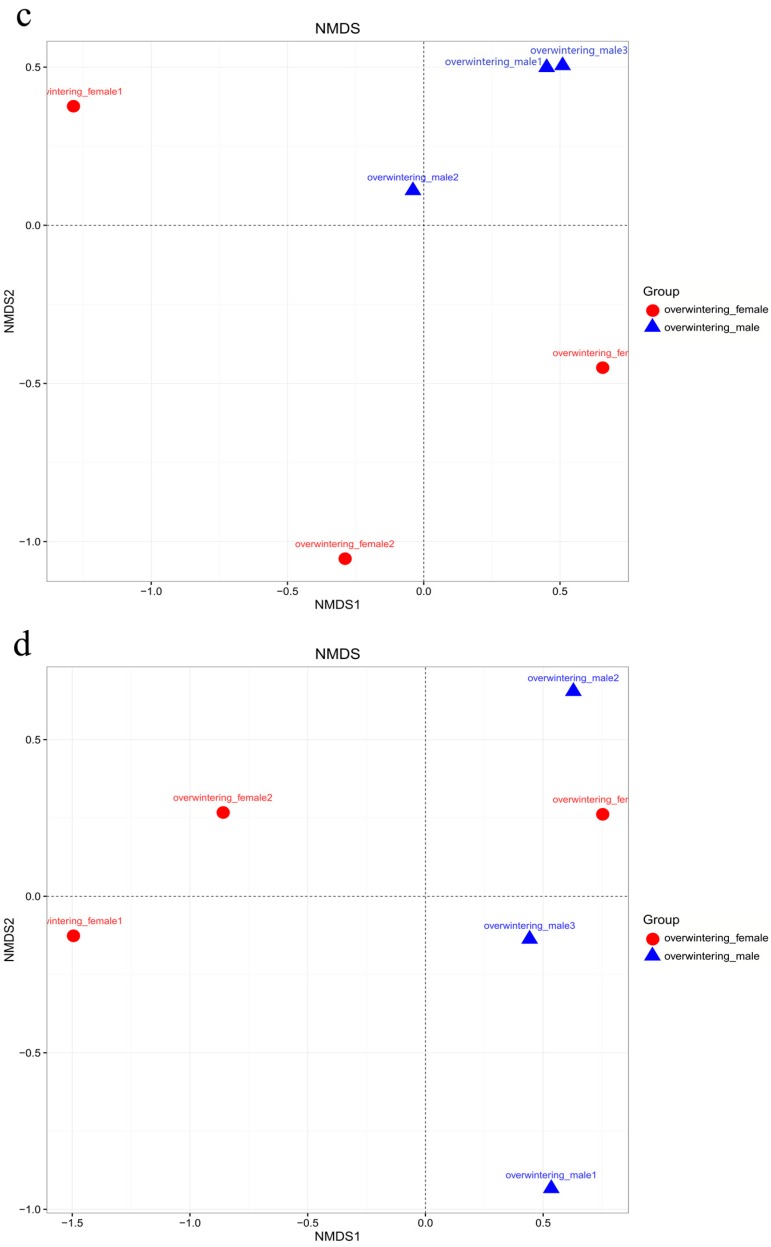
Nonmetric multidimensional scaling analysis of the Bray–Curtis dissimilarity index of the fungal community OTUs (≥97% identity) in different sexes of *T. klimeschi* based on Illumina sequencing of ITS genes. Adult females vs. adult males: (**a**) Unweighted; (**b**) Weighted; overwintering adult females vs. overwintering adult males: (**c**) Unweighted; (**d**) Weighted.

The authors compared community structures between samples using NMDS that revealed the development pattern in different sexes for the unweighted and weighted UniFrac distances (Figure 11). Samples obtained from different sexes of *T. klimeschi* were not scattered relatively within the NMDS plot, indicating that the fungal community structure in *T. klimeschi* is relatively conserved.

#### 2.4.4. Differences Between Samples in Different Sexes

Differences in the community composition among different sexes were tested using the Duncan’s test for multiple comparisons. The community structure of fungi between adult males and females were similar (Figure 10c,d) (See Table A5 in Appendix A).

## 3. Discussion

Although not often obvious to the naked eye, fungi are as deeply enmeshed in the evolutionary history and ecology of life as any other organism on Earth [24]. Furthermore, many data are available for relevant ecological traits such as acting as decomposers by releasing extracellular enzymes to break down various plant biopolymers and using the resulting products [25,26], or communities of endophytic fungi containing wood-decomposer fungi that are present in a latent state prior to plant death [24]. Investigation of the gut fungal community of bark-inhabiting insects is important to better understand the potential role of gut microorganisms in host nutrition, cellulose/hemicellulose degradation, nitrogen fixation, and detoxification processes. Additionally, microbes in the beetle’s intestine have proven to be an important source of enzymes for various industries [27]. The authors not only conduct fungal inventories of *T. klimeschi* across the full host life cycle, but also compare the differences in the community composition in different generations and each sex, which provides new insights into the metabolic potentials of Curculionidae-associated fungal communities. However, not all active fungi could be successfully detected and identified inside the host.

The predominant species found in the intestinal fungal communities of *T. klimeschi* formed a group of low complexity, mainly belonging to the phyla Ascomycota and Basidiomycota. A low level of fungal community complexity is typical of the bark beetle gut discovered to date, except in the fungus-feeding beetles [17,28,29]. The presence and high abundance of these fungal phyla have been previously reported in the gut of larvae from several Coleoptera [30,31,32,33].

However, the structure of the fungal community differed depending on the developmental stages. The fungal community in the neonate larvae was more diverse than the community identified in the mature larvae. Fungal community richness further increased in adults. Since the neonate larvae of *T. klimeschi* feed on inner bark and the gallery contain almost entirely excrement, the living habits were similar to *Trypophloeus striatulus* [34]. Such behavior might contribute to maintaining gut fungal community in the neonate larvae stage. It has been documented that the prepupal larvae of *T. striatulus* evacuate their gut [34]. This phenomenon implies that gut fungal community might be re-structured in the subsequent developmental stages of the life cycle. Furthermore, micro-environments were different between larvae and adults. Moreover, this difference in taxonomic membership might reflect different functional roles across certain life stages. Some fungal taxa guide the entrance point for gallery construction, such as *Trypophloeus striatulus* possibly attraction to odor emitted through lenticels that overlie susceptible *Cytospora*-infested phloem [34].

Fusarium was the most abundant genus and was conserved in all development stages. This result indicates that the conserved fungal community of shared fungal taxa should be well adapted to *T. klimeschi*. It was interesting that Fusarium species (Ascomycota, Nectriaceae) are among the most diverse and widespread plant-infecting fungi, and numerous metabolites produced by Fusarium spp. are toxic to insects [35,36]. The *Tenebrio molitor* larvae were able to use the wheat kernels that were colonized by *Fusarium proliferatumor* and *Fusarium poae* which produced fumonisins, enniatins, and beauvericin during kernel colonization without exhibiting increased mortality. The result suggests that *Tenebrio molitor* can tolerate or metabolize those toxins. Some insect species appear to benefit from the presence of aflatoxin producers [37,38] or mycotoxin produced by Fusarium spp. fungi [39]. The authors have not investigated induced reactions of Fusarium fungi to the presence or feeding of *T. klimeschi*, this will be the subject of subsequent studies.

The genera Nakazawaea, Trichothecium, Aspergillus, Didymella, and Villophora which belonged to the Ascomycota; and Auricularia which belonged to Basidiomycota were more prevalent in the larvae samples.

Nakazawaea is the ascomycete yeast genus, derived from the genus Pichia [40]. Yeasts are frequently isolated from the larvae of bark beetles [41,42]. Additionally, *Nakazawaea* is widely distributed in nature and common to insects that bore into forest trees [43,44,45]. Yeasts are commonly associated with bark beetles and might be an important nutritional source for the insect host [28,46]. Moreover, yeasts such as the *Candida* species can assimilate nutrients such as nitrate, xylose, and cellobiose [47]. Nutritional needs should be different over the developmental stages of the beetle, as the beetle needs nutritional benefits to accomplish different steps in its life style, such as development and ovogenesis [48]. The high prevalence of yeasts associated with the larvae supports the hypothesis that yeasts are essential nutritional elements for the development of the *T*. *klimeschi*. Trichothecium has shown potential for biotransformation and enzyme production [49]. Found in several wood-feeding Coleptera larvae, Trichothecium was the most abundant genus as well [33]. There are few reports about Aspergillus interaction with insects; perhaps it plays the same role as the genus Fusarium. Auricularia are typical wood-inhabiting fungi in the forest ecosystem. They can degrade cellulose, hemicelluloses, and ligini of wood [50]. Some wood-inhabiting fungi provide foods and breeding grounds for some beetles [51,52]. Similar to termites [53], *T. klimeschi* also feeds on plant tissue lacking nitrogen nutrients. Some studies have reported that the mycelium of Auricularia could improve the quality of termite foods and increase egg production by termites [54].

According to this study’s results, the fungal community in the neonate larvae was more diverse than the community identified in other development stages. Considering that the digestive system of the neonate larvae was just maturing, ingesting large amounts of carbon and nitrogen nutrition associated with their symbiotic microorganisms explains why the guts of neonate larvae contained more diverse fungal communities. Overall, these fungi are all relevant to nutritional metabolism. The authors speculate that these fungal symbionts might play important roles in nutrition in *T. klimeschi*.

Adults harbored high proportions of Graphium, which belongs to Ascomycota. The genus Graphium is known as ‘blue stain fungi’ [55]. When bark beetles invade conifers, the fungus taps into the sapwood nitrogen and transports it to the phloem where the beetle feeds, increasing the nitrogen content by up to 40% [56]; this is critical for bark beetle development and survival [2,9,56]. Moreover, one blue stain fungus *Leptographium qinlingensis* is the pathogenic fungus carried by adults of *Dendroctonus armandi*, which develops in the tissues and cells of xylem and phloem of *Pinus armandi* after *D. armandi* attacks healthy host trees, decomposes the secretory resin cells in resin ducts and the parenchyma cell sapwood tissues, then affects the metabolism of resin [57]. The presence of Graphium was also observed in larvae and adult beetles of *Euwallacea fronicatus* as well as in the galleries of several tree species [33,58]. According to the result, the authors speculate that the physiology and biochemistry resistance of *P. alba* var. *pyramidalis* was weakened and nutrient degradation was accelerated by the attacking of genus *Graphium*. Moreover, some fungal taxa guide the entrance point for gallery construction, such as *Trypophloeus striatulus* possibly attraction to odor emitted through lenticels that overlie susceptible *Cytospora*-infested phloem [34]. We speculated that *Graphium* plays an important role in the invasion and colonization of *T. klimeschi*.

The fungal community structures found in the guts of adult females and adult males were similar, suggesting that the fungal community structure in *T. klimeschi* adult is conserved.

Interestingly, there was a significant difference in fungal community structure between the mature larvae and overwintering mature larvae. The pest overwintered in the form of mature larvae. Insects face great challenges in surviving at low temperatures in frigid and temperate zones [59,60,61]. Insects’ cold-tolerance capacity is a dominant factor that affects their adaption to the geographical environment [62,63]. The present results suggested that gut fungal community compositions differed during the overwintering period. Fusarium, which belongs to the Ascomycota, were significantly increased in overwintering mature larvae. Nectriaceae only appeared and was abundant in overwintering mature larvae. We hypothesized that the two genera are associated with the insect overwintering process and resistance to low temperatures.

## 4. Materials and Methods

### 4.1. Collection Site Description

The *T*. *klimeschi* were collected from the bark of infested *Populus alba* var. *pyramidalis* at the shelter belt of Dunhuang City (40°06′50.61″ N, 94°36′10.24″ E), Gansu Province, China. Dunhuang is located in Northwestern Gansu Province, which has a temperate continental, dry climate with low rainfall, high evaporation, large temperature differences between day and night, long sunshine duration, an annual average temperature of 9.4 °C, monthly average maximum temperature of 24.9 °C (July), and monthly average minimum temperature of −9.3% °C (January), extreme maximum temperature of 43.6 °C, and minimum temperature of −28.5 °C.

### 4.2. Life-Cycle of T. klimeschi Description

There were two generations of *T. klimeschi* per year and the pest overwintered in the form of mature larvae. There were two peak periods in a year. Mature larvae began to pupate in early May. Adults started to emerge beginning in mid-May, with a peak from late-May until mid-June. Second generation larvae pupated in mid-July. Adult emergence peaked in August. *T. klimeschi* began wintering in October (see Table A6 in Appendix A).

### 4.3. Insect Collection and Dissection

According to the life history of *T. klimeschi*, larvae and adult females and males were collected from January 2017 to August 2017. The pest overwintered in the form of mature larvae from October to May of the following year. Due to the lowest average temperature being in January, overwintering mature larvae were collected in January 2017. Adults began to emerge in mid-May with a peak from late-May until mid-June. Overwintering adults were collected in May 2017. The neonate larvae of the first generation were collected in June 2017. The mature larvae of the first generation were collected in July 2017. The adults of the first generation were collected in August 2017. To identify gut fungal community structure succession in different development stages, the authors compared neonate larvae, mature larvae, adult females, and adult males. To identify gut fungal community structure succession in different generations, the authors compared adult females with overwintering adult females, adult males with overwintering adult males, and mature larvae with overwintering mature larvae. To identify gut fungal community structure succession in different sexes, the authors compared adult females with adult males and overwintering adult females with overwintering adult males.

The samples were collected at the laboratory in sterile vials. To investigate the influence of sex on gut-associated fungi, adult females and males were separated according to morphology (according to the morphological observation, the salient features distinguishing adult males and females are in the granules of the elytron: the male has three sharp corners on interstria 5 near the tail at the declivity of the left and right elytrons, and the females do not have this feature (relevant data have not been published). A total of 180 insect samples in each life stage were gathered for high-throughput sequencing analysis.

Insect samples were rinsed in sterile water, surface sterilized with 70% ethanol for 3 min, and then rinsed twice in sterile water. Following being placed in 10 mM sterilized phosphate-buffered saline (138 mM NaCl and 2.7 mM KCl, pH 7.4) the insects were dissected under a stereomicroscope with the aid of insect pins to excise the mid-guts and hindguts [64]. Sixty guts were excised from each sample. The treatment in each sample was repeated three times.

### 4.4. DNA Extraction

The E.Z.N.A. Fungal DNA Kit (Omega Biotech, Doraville, GA, USA) was used to extract *T. klimeschi* samples guts fungal DNA following the instruction booklet. The gut fungal DNA was stored at −20 °C before using. DNA samples were mixed in equal concentrations, and the mixed DNA specimens were sent to Personal Biotechnology Co., Ltd. (Shanghai, China) for analysis by high throughput sequencing.

### 4.5. Bioinformatics and Statistical Analysis

Following sequencing, all reads were processed and analyzed using the QIIME package release v1.8.0 [65]. Sequences were clustered by the open-reference OTU clustering using the default settings at a 97% identity threshold. The representative ITS sequences were assigned to taxonomy using the UNITE database [66]. The alpha diversity analysis included observed species, ACE and Chao estimators, Simpson and Shannon diversity indices estimate of coverage. Rarefaction curves were generated based on observed species. According to the OTU classification and classification status identification results, the specific composition of each sample at each classification level was obtained. Nonmetric multidimensional scaling (NMDS) analysis was conducted on the sample-OTU matrix using the Bray–Curtis distances. Additionally, Venn diagrams were also created to observe the partition of the OTUs across different samples. The data differences were analyzed by SPSS (SPSS version 20.0; SPSS, Chicago, IL, USA) software.

## 5. Conclusions

This study revealed the structure of the gut-associated fungal communities in the different developmental stages of *T. klimeschi* and the difference between sexes and two generations. The current study helps gain better understanding of the evolutionary and ecological roles of gut symbionts in many important insect groups. A better understanding of the relationship between fungal symbionts and the Coleoptera host would lead to new concepts and approaches for controlling insect pests by manipulating their microbiota. The authors propose that gut-associated fungi could interfere with the development of *T. klimeschi* and, hence, may have potential as vectors for biocontrol agents.

## Figures and Tables

**Table 1:** Sequences generated before and after quality filter.

Sample	High Quality Sequences	Valid Sequences	High Quality Sequences/Valid Sequences (%)
overwintering mature larvae	41,015 ± 2474	42,304 ± 1921	97
overwintering female adult	46,744 ± 2433	48,111 ± 3367	98
overwintering male adult	42,786 ± 1073	43,667 ± 957	99
neonate larvae	45,228 ± 1564	46,470 ± 1545	98
mature larvae	46,150 ± 1806	46,540 ± 1846	98
female adult	45,388 ± 2334	46,135 ± 2491	99
male adult	43,632 ± 1167	44,336 ± 997	99

**Table 2:** Biodiversity index values of *T. klimeschi* in different development stages.

	Chao 1	ACE	Simpson	Shannon
Neonate larval	354.667 ± 102.627 ^a^	352.667 ± 102.627 ^a^	0.870 ± 0.050 ^a^	4.993 ± 0.532 ^a^
Mature larval	92.333 ± 14.154 ^c^	92.457 ± 13.940 ^c^	0.781 ± 0.001 ^c^	3.331 ± 0.791 ^c^
Female adult	243.667 ± 13.796 ^ab^	240.667 ± 13.796 ^ab^	0.821 ± 0.070 ^ab^	4.032 ± 0.690 ^ab^
Male adult	221.667 ± 76.788 ^b^	221.667 ± 96.308 ^b^	0.800 ± 0.031 ^b^	3.811 ± 0.149 ^b^
*F*	9.293	8.974	4.322	10.136
df	3	3	3	3
*P*	0.008	0.008	0.046	0.007

The data represent the mean ± standard deviation. Different small letters indicated significant difference between sites for that parameter. Means compared using one-way ANOVA, within each group, bars with different letters are significantly different at *p* < 0.05 level.

**Table 3:** Biodiversity index values of *T. klimeschi* in different generations.

	Chao 1	ACE	Simpson	Shannon
Female adult	280.000 ± 12.124	280.000 ± 12.124	0.737 ± 0.034	2.997 ± 0.248
Overwintering female adult	497.027 ± 235.154	497.303 ± 235.623	0.842 ± 0.037	4.940 ± 1.089
*T*	1.675	1.674	2.720	3.352
df	2	2	2	2
*p*	0.236	0.236	0.113	0.079
Male adult	243.000 ± 91.099	243.000 ± 91.099	0.707 ± 0.150	2.897 ± 0.916
Overwintering male adult	407.667 ± 19.757	407.667 ± 19.757	0.862 ± 0.028	4.300 ± 0.335
*T*	2.607	2.607	1.532	1.947
df	2	2	2	2
*p*	0.121	0.121	0.265	0.191
Mature larval	236.320 ± 9.269	239.730 ± 10.922	0.819 ± 0.006	3.230 ± 0.036
Overwintering mature larval	167.333 ± 19.218	167.333 ± 19.218	0.570 ± 0.087	2.240 ± 0.195
*T*	10.643	9.199	5.127	8.606
df	2	2	2	2
*p*	0.009	0.012	0.036	0.013

The data represent the mean ± standard deviation. Means compared using *t*-test.

**Table 4:** Biodiversity index values of *T. klimeschi* in different sexes.

	Chao 1	ACE	Simpson	Shannon
Female adult	432.713 ± 11.738	433.573 ± 12.919	0.792 ± 0.014.	3.670 ± 0.145
Male adult	388.000 ± 103.697	388.000 ± 103.697	0.766 ± 0.119	3.590 ± 0.823
*T*	0.681	0.685	0.358	0.163
df	2	2	2	2
*P*	0.566	0.564	0.755	0.885
Overwintering female adult	452.710 ± 82.078	453.717 ± 88.444	0.878 ± 0.036	5.093 ± 1.154
Overwintering male adult	492.667 ± 70.088	492.667 ± 0.088	0.878 ± 0.046	4.603 ± 0.603
*T*	1.098	1.071	0.016	0.989
df	2	2	2	2
*P*	0.387	0.396	0.989	0.427

The data represent the mean ± standard deviation. Means compared using *t*-test.

## Appendix A

**Table A1:** The relative abundance of the different fungal taxa in different development stages of *T. klimeschi* at the genus level.

Taxonomy		Neonate Larval	Mature Larval	Female Adult	Male Adult	*F*	df	*p*
Phylum	Genus							
Ascomycota	*Fusarium*	0.208 ± 0.188	0.544 ± 0.040	0.267 ± 0.092	0.366 ± 0.240	2.456	3	0.139
	*Nakazawaea*	0.226 ± 0.175a	0.099 ± 0.004ab	0.004 ± 0.003b	0.001 ± 0.001b	4.394	3	0.042
	*Graphium*	-	0.014 ± 0.005b	0.060 ± 0.035a	0.036 ± 0.012ab	5.822	2	0.021
	*Trichothecium*	0.001 ± 0.001b	0.091 ± 0.013a	0.000 ± 0.000b	-	144.986	2	0.000
	*Aspergillus*	0.038 ± 0.015a	0.001 ± 0.000c	0.017 ± 0.005b	0.018 ± 0.005b	10.035	3	0.004
	*Penicillium*	0.010 ± 0.003a	0.001 ± 0.000b	0.011 ± 0.004a	0.011 ± 0.002a	10.614	3	0.004
	*Didymella*	0.001 ± 0.001b	0.007 ± 0.003a	0.002 ± 0.001b	0.002 ± 0.002b	5.452	3	0.025
	*Villophora*	0.008 ± 0.006a	0.000 ± 0.000b	0.001 ± 0.000b	0.001 ± 0.001b	5.110	3	0.029
Basidiomycota	*Auricularia*	0.094 ± 0.071a	0.040 ± 0.001ab	0.013 ± 0.003b	0.017 ± 0.012b	4.072	3	0.050

**Table A2:** The relative abundance of the different fungal taxa in different generations of *T. klimeschi* at the phylum level.

Taxonomy	Female Adult	Overwintering Female Adult	*T*	df	*p*	Male Adult	Overwintering Male Adult	*T*	df	*p*	Mature Larval	Overwintering Mature Larval	*T*	df	*p*
Ascomycota	0.559 ± 0.012	0.649 ± 0.167	0.987	2	0.428	0.637±0.132	0.690 ± 0.112	0.646	2	0.585	0.777 ± 0.040	0.994 ± 0.005	9.457	2	0.011
Basidiomycota	0.032 ± 0.009	0.294 ± 0.131	3.598	2	0.069	0.036 ± 0.025	0.198 ± 0.152	2.004	2	0.183	0.013±0.007	0.003 ± 0.002	4.000	2	0.057

**Table A3:** The relative abundance of the different fungal taxa in different generation of *T. klimeschi* at the genus level.

**(A)**													
**Taxonomy**		**Female Adult**	**Overwintering Female Adult**	***T***	**df**	***p***		**Male Adult**		**Overwintering Male Adult**	***T***	**df**	***p***
**Phylum**	**Genus**												
Ascomycota	*Fusarium*	0.267 ± 0.092	0.025 ± 0.022	4.254	2	0.051		0.366 ± 0.240		0.097 ± 0.131	4.196	2	0.052
	*Penicillium*	0.011 ± 0.004	0.245 ± 0.196	2.098	2	0.171		0.011 ± 0.02		0.240 ± 0.196	2.043	2	0.178
	*Acremonium*	0.001 ± 0.000	0.003 ± 0.004	1.037	2	0.409		0.001 ± 0.000		0.082 ± 0.123	1.155	2	0.367
	*Aspergillus*	0.017 ± 0.005	0.054 ± 0.036	1.546	2	0.262		0.018 ± 0.005		0.026 ± 0.005	6.003	2	0.027
	*Graphium*	0.060 ± 0.035	0.001 ± 0.002	2.817	2	0.106		0.036 ± 0.012		0.003 ± 0.005	3.455	2	0.075
Basidiomycota	*Quambalaria*	0.000 ± 0.000	0.033 ± 0.036	1.534	2	0.265		0.000 ± 0.000		0.083 ± 0.131	1.091	2	0.389
	*Auricularia*	0.013 ± 0.003	0.201 ± 0.152	2.144	2	0.165		0.017 ± 0.012		0.070 ± 0.026	3.305	2	0.081
**(B** **)**													
**Taxonomy**		**Mature Larval**	**Overwintering Mature Larval**	***T***			**df**		***p***				
**Phylum**	**Genus**												
Ascomycota	*Fusarium*	0.544 ± 0.040	0.828 ± 0.148	4.352			2		0.051				
	*Nakazawaea*	0.099 ± 0.004	0.013 ± 0.010	18.758			2		0.171				
	*Graphium*	0.014 ± 0.005	0.087 ± 0.106	1.137			2		0.409				
	*Trichothecium*	0.091 ± 0.013	-	-			-		-				
	Unidentified_Nectriaceae	-	0.056 ± 0.047	-			-		-				
	*Cryptosphaeria*	0.010 ± 0.010	-	-			-		-				

**Table A4:** The relative abundance of the different fungal taxa in different sexes of *T. klimeschi* at the phylum level.

Taxonomy	Female Adult	Male Adult	*T*	df	*p*	Overwintering Female Adult	Overwintering Male Adult	*T*	df	*p*
Ascomycota	0.559 ± 0.012	0.636 ± 0.133	1.057	2	0.401	0.648 ± 0.170	0.691 ± 0.113	0.780	2	0.517
Basidiomycota	0.032 ± 0.009	0.035 ± 0.025	0.260	2	0.819	0.295 ± 0.132	0.198 ± 0.153	0.934	2	0.449

**Table A5:** The relative abundance of the different fungal taxa in different sexes of *T. klimeschi* at the genus level.

Taxonomy		Female Adult	Male Adult	*T*	df	*p*	Overwintering Female Adult	Overwintering Male Adult	*T*	df	*p*
Phylum	Genus										
Ascomycota	*Fusarium*	0.267 ± 0.092	0.366 ± 0.240	0.566	2	0.629	0.025 ± 0.022	0.097 ± 0.131	1.148	2	0.370
	*Aspergillus*	0.017 ± 0.005	0.018 ± 0.005	0.212	2	0.851	0.054 ± 0.036	0.026 ± 0.005	1.514	2	0.269
	*Penicillium*	0.011 ± 0.004	0.010 ± 0.002	0.164	2	0.885	0.245 ± 0.196	0.240 ± 0.196	0.123	2	0.913
	*Graphium*	0.060 ± 0.035	0.036 ± 0.012	0.322	2	0.798	0.001 ± 0.002	0.003 ± 0.005	0.164	2	0.885
	*Acremonium*	0.001 ± 0.000	0.001 ± 0.000	0.132	2	0.911	0.003 ± 0.004	0.082 ± 0.123	1.101	2	0.386
Basidiomycota	*Auricularia*	0.013 ± 0.003	0.017 ± 0.012	0.580	2	0.620	0.201 ± 0.152	0.070 ± 0.026	1.514	2	0.269
	*Quambalaria*	0.000 ± 0.000	0.000 ± 0.000	0.126	2	0.928	0.033 ± 0.036	0.083 ± 0.131	0.600	2	0.609

**Table A6:** The life cycle of *T. klimeschi.*

Month	Jan.–Apr.			Mar.			Jun.			Jul.			Aug.			Sep.			Oct.			Nov.–Dec.		
	E	M	L	E	M	L	E	M	L	E	M	L	E	M	L	E	M	L	E	M	L	E	M	L
Overwintering generation	▲	▲	▲	▲																				
				−	−	−																		
					+	+	+	+	+															
First generation							○	○	○															
								▲	▲	▲	▲	▲	▲	▲										
											−	−	−	−										
												+	+	+	+	+								
Overwintering generation														○	○	○	○							
															▲	▲	▲	▲	▲	▲	▲	▲	▲	▲

Remarks: +: Adult; ○: Egg; ▲: Larva; −: Pupa; E: Early; M: Middle; L: late.
